# Using molecular principal axes for structural comparison: determining the tertiary changes of a FAB antibody domain induced by antigenic binding

**DOI:** 10.1186/1472-6807-7-77

**Published:** 2007-11-09

**Authors:** B David Silverman

**Affiliations:** 1 IBM Thomas J. Watson Research Center P. O. Box 218, Yorktown Heights, NY 10598, USA

## Abstract

**Background:**

Comparison of different protein x-ray structures has previously been made in a number of different ways; for example, by visual examination, by differences in the locations of secondary structures, by explicit superposition of structural elements, e.g. *α*-carbon atom locations, or by procedures that utilize a common symmetry element or geometrical feature of the structures to be compared.

**Results:**

A new approach is applied to determine the structural changes that an antibody protein domain experiences upon its interaction with an antigenic target. These changes are determined with the use of two different, however comparable, sets of principal axes that are obtained by diagonalizing the second-order tensors that yield the moments-of-geometry as well as an ellipsoidal characterization of domain shape, prior to and after interaction. Determination of these sets of axes for structural comparison requires no internal symmetry features of the domains, depending solely upon their representation in three-dimensional space. This representation may involve atomic, C*α*, or residue centroid coordinates. The present analysis utilizes residue centroids. When the structural changes are minimal, the principal axes of the domains, prior to and after interaction, are essentially comparable and consequently may be used for structural comparison. When the differences of the axes cannot be neglected, but are nevertheless slight, a smaller relatively invariant substructure of the domains may be utilized for comparison. The procedure yields two distance metrics for structural comparison. First, the displacements of the residue centroids due to antigenic binding, referenced to the ellipsoidal principal axes, are noted. Second, changes in the ellipsoidal distances with respect to the non-interacting structure provide a direct measure of the spatial displacements of the residue centroids, towards either the interior or exterior of the domain.

**Conclusion:**

With use of x-ray data from the protein data bank (PDB), these two metrics are shown to highlight, in a manner different from before, the structural changes that are induced in the overall domains as well as in the H3 loops of the complementarity-determining regions (CDR) upon FAB antibody binding to a truncated and to a synthetic hemagglutinin viral antigenic target.

## Background

Comparison of different protein x-ray structures has previously been made in a number of different ways; for example, by visual examination, by differences in the locations of secondary structures, by explicit superposition of structural elements, e.g. *α*-carbon atom locations, or by procedures that utilize a common symmetry element or geometrical feature of the structures to be compared. This latter procedure has been utilized in connection with the identification of the structurally conserved residues within the core of the immunoglobulin variable domains [1]. A singular advantage of such procedure, compared with the other procedures, is that it provides additional information that relates the location of the residues to attributes of the geometrical feature to which these locations have been aligned or referenced. For example, it has been pointed out that the alignment, based on the pseudo 2-fold symmetry axis of the variable domains of known immunoglobulin structures, *provides information about the possible structural or functional roles of residues *(italics quoted verbatim in the reference) [2].

The overall shape or distribution of the amino acid residues of a protein domain may also be considered a geometric invariant of a set of structures undergoing comparison when the differences in their global geometries are small and involve only a minor fraction of the residues comprising the domains. The representation of such shape may be given by the distribution of atomic, C*α*, or residue centroid locations in three-dimensional space. Such representation, generating an ellipsoidal characterization of the shape of a domain, had previously provided useful information in connection with drug discovery [3] and with the spatial distribution of residue hydrophobicity within protein domains [4]. This characterization of domain shape provides two spatial metrics, one of which references the location of a residue to the ellipsoidal principal axes of the domain and the other which yields information detailing the proximity of a residue to either the interior or exterior of the protein domain. The present paper describes how the changes in antibody structure that occur upon binding to an antigenic target are characterized by the consequent changes of these two metrics

One limitation of the present procedure is that the unliganded antibody structure (domain) is required as well as its antibody structure (domain) in the complex. While the Protein Data Bank (PDB) [5] has numerous antibodies complexed with their viral or chemical targets, there are many fewer unliganded structures listed. The number of PDB structures satisfying our requirements is further reduced since interest will be focused on antibody binding to an influenza viral hemagglutinin antigenic target. Furthermore, we require 100% sequence identity between the unliganded and complexed heavy and light chain domains of the FAB (antibody fragment). Two PDB antibody structures that satisfy these requirements are antibody HC19 complexed with a truncated hemagglutinin structure [6], PDB id 2VIR, and its unliganded antibody structure [7], PDB id 1GIG; and FAB 17/9 complexed with a peptide hemagglutinin mimetic [8], PDB id 1IFH, and its unliganded antibody structure [9], pdb id 1HIL. Interest will focus on the two distance metrics of the ellipsoidal characterization of protein domain structure and on the complementary information they present that describes the structural changes that occur upon the antigenic binding of these two antibodies. Hopefully, such information involving a different perspective from that provided previously may assist in the *attempts to design synthetic vaccines on the basis of X-ray structures of anti-body-peptide complexes *[8].

## Methods

The ellipsoidal characterization of a protein domain has been previously described [10], however, it will be useful to indulge in a degree of redundancy to smoothly illustrate the appropriate extension required for the present application. The present calculations are based upon the residue side-chain centroids of the protein. However, as mentioned previously the distribution of points in three-dimensional space chosen to represent a protein structure may well be that of the C*α *coordinates, of the atomic coordinates, or of any other set of points in space chosen to detail protein structure.

The residue centroids are calculated with inclusion of only the heavy atoms of the side-chain and without the backbone atoms. One could have included the backbone atoms as well in calculating the residue centroids which would yield minor modifications of the present results. Not including the backbone atoms places the residue centroids at a greater distance from the backbone and provides somewhat greater emphasis with regard to differences in side chain location and orientation.

The distribution of residue centroid side-chain locations, r→i
 MathType@MTEF@5@5@+=feaafiart1ev1aaatCvAUfKttLearuWrP9MDH5MBPbIqV92AaeXatLxBI9gBaebbnrfifHhDYfgasaacPC6xNi=xH8viVGI8Gi=hEeeu0xXdbba9frFj0xb9qqpG0dXdb9aspeI8k8fiI+fsY=rqGqVepae9pg0db9vqaiVgFr0xfr=xfr=xc9adbaqaaeGacaGaaiaabeqaaeqabiWaaaGcbaGafmOCaiNbaSaadaWgaaWcbaGaemyAaKgabeaaaaa@2ED9@, in three-dimensional space enables the assignment of a "center-of-the-protein", r→c
 MathType@MTEF@5@5@+=feaafiart1ev1aaatCvAUfKttLearuWrP9MDH5MBPbIqV92AaeXatLxBI9gBaebbnrfifHhDYfgasaacPC6xNi=xH8viVGI8Gi=hEeeu0xXdbba9frFj0xb9qqpG0dXdb9aspeI8k8fiI+fsY=rqGqVepae9pg0db9vqaiVgFr0xfr=xfr=xc9adbaqaaeGacaGaaiaabeqaaeqabiWaaaGcbaGafmOCaiNbaSaadaWgaaWcbaGaem4yamgabeaaaaa@2ECD@, namely, as the centroid of all protein residue side-chain centroids:

r→c=1n∑ir→i
 MathType@MTEF@5@5@+=feaafiart1ev1aaatCvAUfKttLearuWrP9MDH5MBPbIqV92AaeXatLxBI9gBaebbnrfifHhDYfgasaacPC6xNi=xI8qiVKYPFjYdHaVhbbf9v8qqaqFr0xc9vqFj0dXdbba91qpepeI8k8fiI+fsY=rqGqVepae9pg0db9vqaiVgFr0xfr=xfr=xc9adbaqaaeGacaGaaiaabeqaaeqabiWaaaGcbaGafmOCaiNbaSaadaWgaaWcbaGaem4yamgabeaakiabg2da9KqbaoaalaaabaGaeGymaedabaGaemOBa4gaaOWaaabuaeaacuWGYbGCgaWcamaaBaaaleaacqWGPbqAaeqaaaqaaiabdMgaPbqab0GaeyyeIuoaaaa@39A1@

*n *is the total number of residues.

The ellipsoidal representation of protein domain shape is obtained by diagonalizing the second-order moments-of-geometry tensor, G˜
 MathType@MTEF@5@5@+=feaafiart1ev1aaatCvAUfKttLearuWrP9MDH5MBPbIqV92AaeXatLxBI9gBaebbnrfifHhDYfgasaacPC6xNi=xH8viVGI8Gi=hEeeu0xXdbba9frFj0xb9qqpG0dXdb9aspeI8k8fiI+fsY=rqGqVepae9pg0db9vqaiVgFr0xfr=xfr=xc9adbaqaaeGacaGaaiaabeqaaeqabiWaaaGcbaGafm4raCKbaGaaaaa@2CF9@, which consists of the following elements.

G˜=∑i(1˜|r→i−r→c|2−(r→i−r→c)(r→i−r→c))
 MathType@MTEF@5@5@+=feaafiart1ev1aaatCvAUfKttLearuWrP9MDH5MBPbIqV92AaeXatLxBI9gBaebbnrfifHhDYfgasaacPC6xNi=xI8qiVKYPFjYdHaVhbbf9v8qqaqFr0xc9vqFj0dXdbba91qpepeI8k8fiI+fsY=rqGqVepae9pg0db9vqaiVgFr0xfr=xfr=xc9adbaqaaeGacaGaaiaabeqaaeqabiWaaaGcbaGafm4raCKbaGaacqGH9aqpdaaeqbqaaiabcIcaOiqbigdaXyaaiaWaaqWaaeaacuWGYbGCgaWcamaaBaaaleaacqWGPbqAaeqaaOGaeyOeI0IafmOCaiNbaSaadaWgaaWcbaGaem4yamgabeaaaOGaay5bSlaawIa7aaWcbaGaemyAaKgabeqdcqGHris5aOWaaWbaaSqabeaacqaIYaGmaaGccqGHsislcqGGOaakcuWGYbGCgaWcamaaBaaaleaacqWGPbqAaeqaaOGaeyOeI0IafmOCaiNbaSaadaWgaaWcbaGaem4yamgabeaakiabcMcaPiabcIcaOiqbdkhaYzaalaWaaSbaaSqaaiabdMgaPbqabaGccqGHsislcuWGYbGCgaWcamaaBaaaleaacqWGJbWyaeqaaOGaeiykaKIaeiykaKcaaa@5225@

Where 1˜
 MathType@MTEF@5@5@+=feaafiart1ev1aaatCvAUfKttLearuWrP9MDH5MBPbIqV92AaeXatLxBI9gBaebbnrfifHhDYfgasaacPC6xNi=xH8viVGI8Gi=hEeeu0xXdbba9frFj0xb9qqpG0dXdb9aspeI8k8fiI+fsY=rqGqVepae9pg0db9vqaiVgFr0xfr=xfr=xc9adbaqaaeGacaGaaiaabeqaaeqabiWaaaGcbaGafGymaeJbaGaaaaa@2CD2@ is the unit dyadic.

The moments-of-geometry tensor is analogous to the moments-of-inertia tensor, however, with each point assigned a mass of one. The diagonalization of G˜
 MathType@MTEF@5@5@+=feaafiart1ev1aaatCvAUfKttLearuWrP9MDH5MBPbIqV92AaeXatLxBI9gBaebbnrfifHhDYfgasaacPC6xNi=xH8viVGI8Gi=hEeeu0xXdbba9frFj0xb9qqpG0dXdb9aspeI8k8fiI+fsY=rqGqVepae9pg0db9vqaiVgFr0xfr=xfr=xc9adbaqaaeGacaGaaiaabeqaaeqabiWaaaGcbaGafm4raCKbaGaaaaa@2CF9@ provides the moments-of-geometry, *g*_1_, *g*_2_, and *g*_3_.

The moments provide an ellipsoidal characterization of protein shape.

g1xp2+g2yp2+g3zp2=d2
 MathType@MTEF@5@5@+=feaafiart1ev1aaatCvAUfKttLearuWrP9MDH5MBPbIqV92AaeXatLxBI9gBaebbnrfifHhDYfgasaacPC6xNi=xI8qiVKYPFjYdHaVhbbf9v8qqaqFr0xc9vqFj0dXdbba91qpepeI8k8fiI+fsY=rqGqVepae9pg0db9vqaiVgFr0xfr=xfr=xc9adbaqaaeGacaGaaiaabeqaaeqabiWaaaGcbaGaem4zaC2aaSbaaSqaaiabigdaXaqabaGccqWG4baEdaqhaaWcbaGaemiCaahabaGaeGOmaidaaOGaey4kaSIaem4zaC2aaSbaaSqaaiabikdaYaqabaGccqWG5bqEdaqhaaWcbaGaemiCaahabaGaeGOmaidaaOGaey4kaSIaem4zaC2aaSbaaSqaaiabiodaZaqabaGccqWG6bGEdaqhaaWcbaGaemiCaahabaGaeGOmaidaaOGaeyypa0Jaemizaq2aaWbaaSqabeaacqaIYaGmaaaaaa@44FF@

The *x*_*p*_, *y*_*p*_, *z*_*p*_, are the coordinates in the frame of the ellipsoidal principal axes with the centroid of the structure as origin. If the magnitudes are ordered as,

*g*_1 _<*g*_2 _<*g*_3 _

the major semi-principal axis is of length, *d*/*g*_1_^1/2^.

Each *i *th residue at location, *x*_*ip*_, *y*_*ip*_, *z*_*ip*_, in the principal axis frame, can be considered to reside on an ellipsoid with major semi-principal axis of length, *d*_*i*_/*g*_1_^1/2^, namely,

g1xip2+g2yip2+g3zip2=di2
 MathType@MTEF@5@5@+=feaafiart1ev1aaatCvAUfKttLearuWrP9MDH5MBPbIqV92AaeXatLxBI9gBaebbnrfifHhDYfgasaacPC6xNi=xI8qiVKYPFjYdHaVhbbf9v8qqaqFr0xc9vqFj0dXdbba91qpepeI8k8fiI+fsY=rqGqVepae9pg0db9vqaiVgFr0xfr=xfr=xc9adbaqaaeGacaGaaiaabeqaaeqabiWaaaGcbaGaem4zaC2aaSbaaSqaaiabigdaXaqabaGccqWG4baEdaqhaaWcbaGaemyAaKMaemiCaahabaGaeGOmaidaaOGaey4kaSIaem4zaC2aaSbaaSqaaiabikdaYaqabaGccqWG5bqEdaqhaaWcbaGaemyAaKMaemiCaahabaGaeGOmaidaaOGaey4kaSIaem4zaC2aaSbaaSqaaiabiodaZaqabaGccqWG6bGEdaqhaaWcbaGaemyAaKMaemiCaahabaGaeGOmaidaaOGaeyypa0Jaemizaq2aa0baaSqaaiabdMgaPbqaaiabikdaYaaaaaa@4A6B@

For a compact globular protein, the residue with the largest *d*_*i *_can specify the ellipsoid defining a presumed protein surface. Residues with the same *d*_*i*_, namely, residues residing on the same ellipsoid are at the same radial fractional distance from the protein centroid to the protein ellipsoidal surface. Rewriting equation 5 as:

xip2+g′2yip2+g′3zip2=d′i2
 MathType@MTEF@5@5@+=feaafiart1ev1aaatCvAUfKttLearuWrP9MDH5MBPbIqV92AaeXatLxBI9gBaebbnrfifHhDYfgasaacPC6xNi=xI8qiVKYPFjYdHaVhbbf9v8qqaqFr0xc9vqFj0dXdbba91qpepeI8k8fiI+fsY=rqGqVepae9pg0db9vqaiVgFr0xfr=xfr=xc9adbaqaaeGacaGaaiaabeqaaeqabiWaaaGcbaGaemiEaG3aa0baaSqaaiabdMgaPjabdchaWbqaaiabikdaYaaakiabgUcaRiqbdEgaNzaafaWaaSbaaSqaaiabikdaYaqabaGccqWG5bqEdaqhaaWcbaGaemyAaKMaemiCaahabaGaeGOmaidaaOGaey4kaSIafm4zaCMbauaadaWgaaWcbaGaeG4mamdabeaakiabdQha6naaDaaaleaacqWGPbqAcqWGWbaCaeaacqaIYaGmaaGccqGH9aqpcuWGKbazgaqbamaaDaaaleaacqWGPbqAaeaacqaIYaGmaaaaaa@4812@

with

g′2=g2/g1;g′3=g3/g1;d′i2=di2/g1
 MathType@MTEF@5@5@+=feaafiart1ev1aaatCvAUfKttLearuWrP9MDH5MBPbIqV92AaeXatLxBI9gBaebbnrfifHhDYfgasaacPC6xNi=xI8qiVKYPFjYdHaVhbbf9v8qqaqFr0xc9vqFj0dXdbba91qpepeI8k8fiI+fsY=rqGqVepae9pg0db9vqaiVgFr0xfr=xfr=xc9adbaqaaeGacaGaaiaabeqaaeqabiWaaaGcbaGafm4zaCMbauaadaWgaaWcbaGaeGOmaidabeaakiabg2da9iabdEgaNnaaBaaaleaacqaIYaGmaeqaaOGaei4la8Iaem4zaC2aaSbaaSqaaiabigdaXaqabaGccqGG7aWocuWGNbWzgaqbamaaBaaaleaacqaIZaWmaeqaaOGaeyypa0Jaem4zaC2aaSbaaSqaaiabiodaZaqabaGccqGGVaWlcqWGNbWzdaWgaaWcbaGaeGymaedabeaakiabcUda7iqbdsgaKzaafaWaa0baaSqaaiabdMgaPbqaaiabikdaYaaakiabg2da9iabdsgaKnaaDaaaleaacqWGPbqAaeaacqaIYaGmaaGccqGGVaWlcqWGNbWzdaWgaaWcbaGaeGymaedabeaaaaa@4D1C@

enables d′i
 MathType@MTEF@5@5@+=feaafiart1ev1aaatCvAUfKttLearuWrP9MDH5MBPbIqV92AaeXatLxBI9gBaebbnrfifHhDYfgasaacPC6xNi=xH8viVGI8Gi=hEeeu0xXdbba9frFj0xb9qqpG0dXdb9aspeI8k8fiI+fsY=rqGqVepae9pg0db9vqaiVgFr0xfr=xfr=xc9adbaqaaeGacaGaaiaabeqaaeqabiWaaaGcbaGafmizaqMbauaadaWgaaWcbaGaemyAaKgabeaaaaa@2EB7@ to be used as a measure of the radial fractional distance of the *i*th residue from the center of the protein to the protein surface. This distance, which will be called the ellipsoidal distance, is used in the calculations. It is just the value of the semi-principal major axis of the ellipsoid upon which the residue centroid is found. It provides a more accurate characterization of the amino acid proximity to the protein exterior than the radial distance from the protein center to the residue centroid, as well as providing a distance that correlates more closely with residue solvent accessibility [11].

To calculate the displacements of the residues in the liganded compared with the unliganded structure, the calculations are performed twice; once inclusive of all residues of the unliganded domain which we will designate by "a" and once inclusive of all residues of the domain in the complex which we will designate by "b".

The magnitude of the displacement of the *i*th residue centroid of the complexed domain with respect to its location in the unliganded domain, *D*_*i*_, is given by the distance between the coordinates of the centroids with respect to the two different sets of principal axes.

*D*_*i *_= [(*x*_*bip *_- *x*_*aip*_)^2 ^+ (*y*_*bip *_- *y*_*aip*_)^2 ^+ (*z*_*bip *_- *z*_*aip*_)^2^]^1/2 ^

The subscript, with either an "a" or "b", designates whether the coordinate is referenced to the principal axes of the unliganded or of the liganded domain, respectively.

The difference or the change in the ellipsoidal distance of the *i*th residue, *E*_*i*_, is given by:

Ei=d′bi−d′ai
 MathType@MTEF@5@5@+=feaafiart1ev1aaatCvAUfKttLearuWrP9MDH5MBPbIqV92AaeXatLxBI9gBaebbnrfifHhDYfgasaacPC6xNi=xI8qiVKYPFjYdHaVhbbf9v8qqaqFr0xc9vqFj0dXdbba91qpepeI8k8fiI+fsY=rqGqVepae9pg0db9vqaiVgFr0xfr=xfr=xc9adbaqaaeGacaGaaiaabeqaaeqabiWaaaGcbaGaemyrau0aaSbaaSqaaiabdMgaPbqabaGccqGH9aqpcuWGKbazgaqbamaaBaaaleaacqWGIbGycqWGPbqAaeqaaOGaeyOeI0IafmizaqMbauaadaWgaaWcbaGaemyyaeMaemyAaKgabeaaaaa@3922@

When the difference between the antibody structures of the liganded and unliganded domains is minimal this procedure will provide a relatively accurate characterization of the displacements and changes in the ellipsoidal distances that occur. However, if the liganded and unliganded structures differ sufficiently, the calculated differences may then be anomalous. For example residues far from the binding site should exhibit minimal displacements upon complexing. If this is not observed then the liganded and unliganded structures would be sufficiently different and not provide principal axes that are comparable and consequently appropriate to be used for structural comparison. If, however, only a minor region or part of the liganded and unliganded structures differs, e.g., perhaps only differing in the vicinity of the binding site, such difficulty may be circumvented by the choice of comparable substructures to reference the displacements and the changes in ellipsoidal distances. The substructures chosen, for example, may involve the elimination of residues that exhibit significant displacements between the liganded and unliganded structures. In pursuit of such strategy, after diagonalization of the tensor, all residue locations of the substructures will be provided; however, locations of the residues that have been eliminated in the choice of the substructures would then have to be calculated by translating the location of these residues to the centers-of-geometry of each of the substructures and then by rotating into the orientation of the principal axes of the substructures. This procedure will be demonstrated in the comparison between the residue locations of the 1IFH and 1HIL pdb viral structures.

Finally, it should be noted that this strategy of referencing structures undergoing comparison to the sets of principal axes of relatively invariant substructures represents a more general and inclusive strategy than referencing the structures to sets of symmetry axes, e.g., alignments based on the pseudo 2-fold symmetry axes of the variable domains of known immunoglobulin structures. In the present case the invariance of the axes is a consequence of the invariance of the substructures and need not be related to any explicit structural symmetry.

## Results and discussion

**Figure 1:**
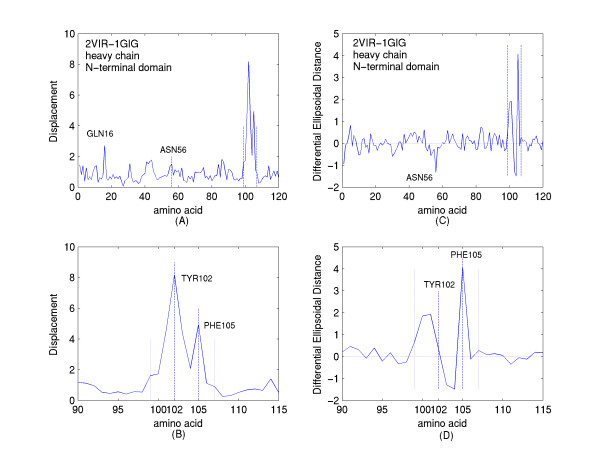
A. The Displacements in Angstroms of the amino acid centroids from their locations in the unliganded domain of the heavy chain (PDB file, 1GIG) to their locations in the complexed domain of the heavy chain (PDB file, 2VIR). B. The Displacements described in A, shown on an expanded scale about the amino acid residues of greatest Displacement. C. The differences in the ellipsoidal distances (Differential Ellipsoidal Distances) in Angstroms of the amino acid centroids of the heavy chain domain of the complex (PDB, 2VIR) from their values in the uncomplexed heavy chain domain (PDB, 1GIG). D. The differences described in C, shown on an expanded scale about the amino acid residues showing the greatest differences. The units of displacements and distances are also in Angstroms in all subsequent figures.

**Figure 2:**
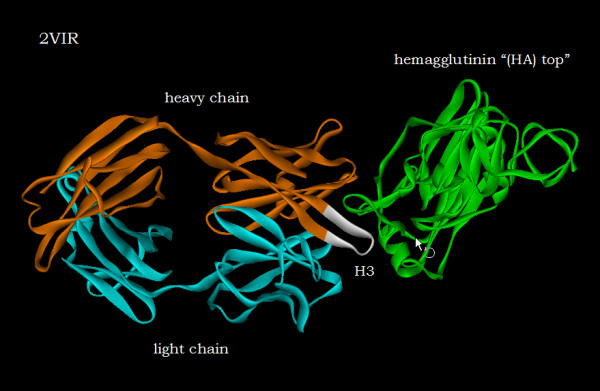
The H3 CDR (complementarity-determining region) region of the heavy chain, highlighted in white, which undergoes the most extensive structural modification upon antibody binding to the truncated "HA top".

**Figure 3:**
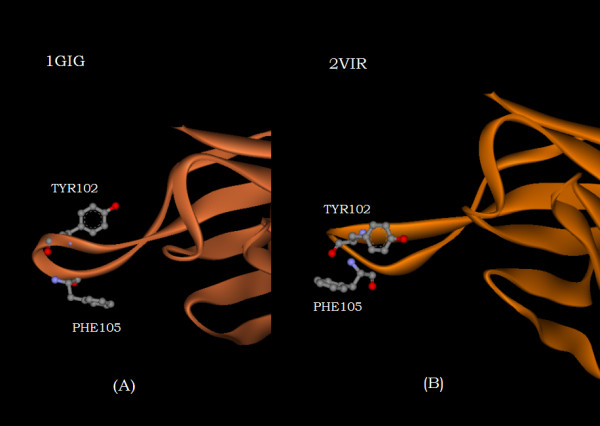
The orientation of the heavy chain residues TYR102 and PHE105 in the uncomplexed structure (1GIG) (figure 3A) and in the complexed structure (2VIR) (figure (3B), respectively, with respect to the bulk of the heavy chain (off and to the right of the figure).

Calculations have been performed utilizing the x-ray structure of a free HC19 FAB [7; pdb id 1GIG] and the structure of the HC19 FAB in complex with the membrane distal domain of X31 hemagglutinin ('HA-top') [6; pdb id 2VIR]. Figures 1A and 1C show the displacements and differences in ellipsoidal distances in Angstroms of the amino acid centroids of the N-terminal FAB heavy chain domain of the complex, from their locations in the free or unliganded antibody domain. First, one notes, that aside from the regions of amino acids that are bracketed by the dashed lines or specifically labeled, the displacements and differences are small, mainly less than 1 Angstrom, confirming that a major portion of the overall antibody structure, before and after binding is comparable. The bracketed region spans residues PHE99 to TYR107. This region, the region undergoing the most extensive structural modification of the antibody upon binding to the truncated "HA top" involves the tip of the H3 CDR (complementarity-determining region). It is highlighted in white in figure 2. Figures 1B and 1D, which are expanded views of the bracketed regions, accentuate the complementarity of the information provided by the two different distance metrics. Note that the amino acid with the greatest displacement, TYR102 in figure 1B, shows a difference in ellipsoidal distance in figure 1D that is approximately equal to zero; whereas PHE105, which has a displacement less than TYR102 exhibits the largest value of differential ellipsoidal distance. Figure 3 illustrates the reason for this difference. Comparison of figure 3A with figure 3b shows that the structural modification of the H3 loop upon binding involves the swapping of the location of TYR102, behind the loop shown in the unliganded structure of figure 3A, to a location in front of the loop in the liganded complex shown in figure 3B. While this involves a relatively large displacement from its position in the unliganded structure, its distance from the interior of the heavy chain domain (to the right in the figures) is relatively unchanged. This contrasts with the rotation of the PHE105 six-membered ring which clearly places its residue centroid upon complexation at a greater distance from the interior of the heavy chain domain.

**Figure 4:**
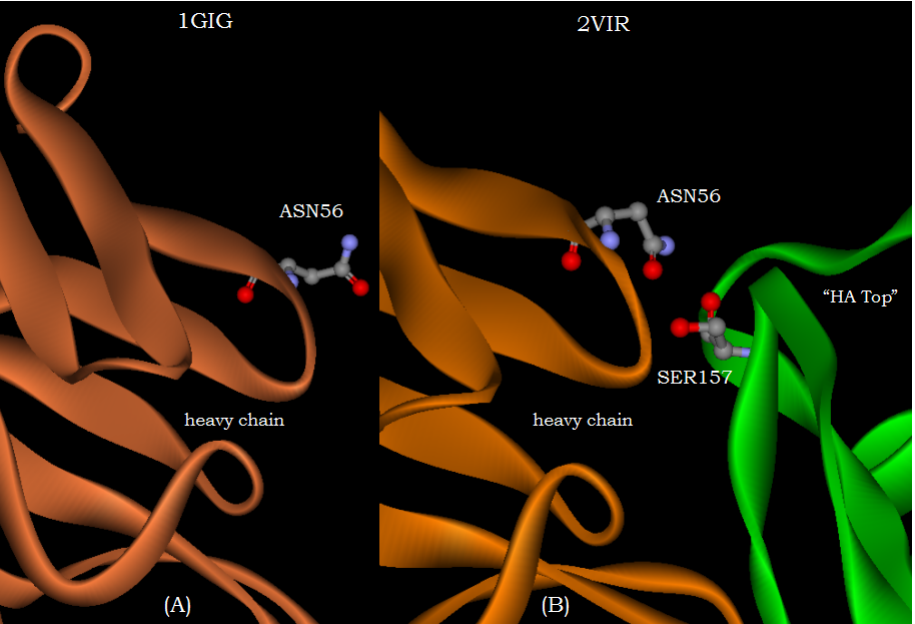
The orientation of the residue ASN56 of the heavy chain prior to complexing (Figure 4A) with its orientation in interaction with the SER157 residue of the "HA top" (Figure 4B) after complexing.

Furthermore, while figure 1A shows the ASN56 residue of the H2 CDR loop of the N-terminal domain of the heavy chain to exhibit only a slightly greater displacement than the displacements of its adjacent residues, figure 1C shows its comparative displacement towards the interior of the N-terminal domain of the heavy chain to be enhanced compared with those of its neighbors. This is apparently mediated by the interaction in the complex between ASN56 and its proximate neighbor SER157 of the "HA-top" as shown in figure 4.

Moments of the 1GIG and 2VIR structures obtained by the diagonalization of equation 2 differ by a few percent and the two sets of principal coordinates yield coordinate frames with axes alignments that differ by at most several degrees.

Certain enhanced displacements apparently identify residues that have been spatially shifted due to crystal packing. Figure 1A shows an enhanced displacement, with respect to the local background, of the residue GLN16 of the heavy chain N-terminal domain. Such displacement, clearly unrelated to antibody binding, appears to arise from crystal packing. Residues significantly displaced, while not in the vicinity of the region of binding and also observed to be considerably solvent exposed in the free state of the antibody can be so identified.

**Figure 5:**
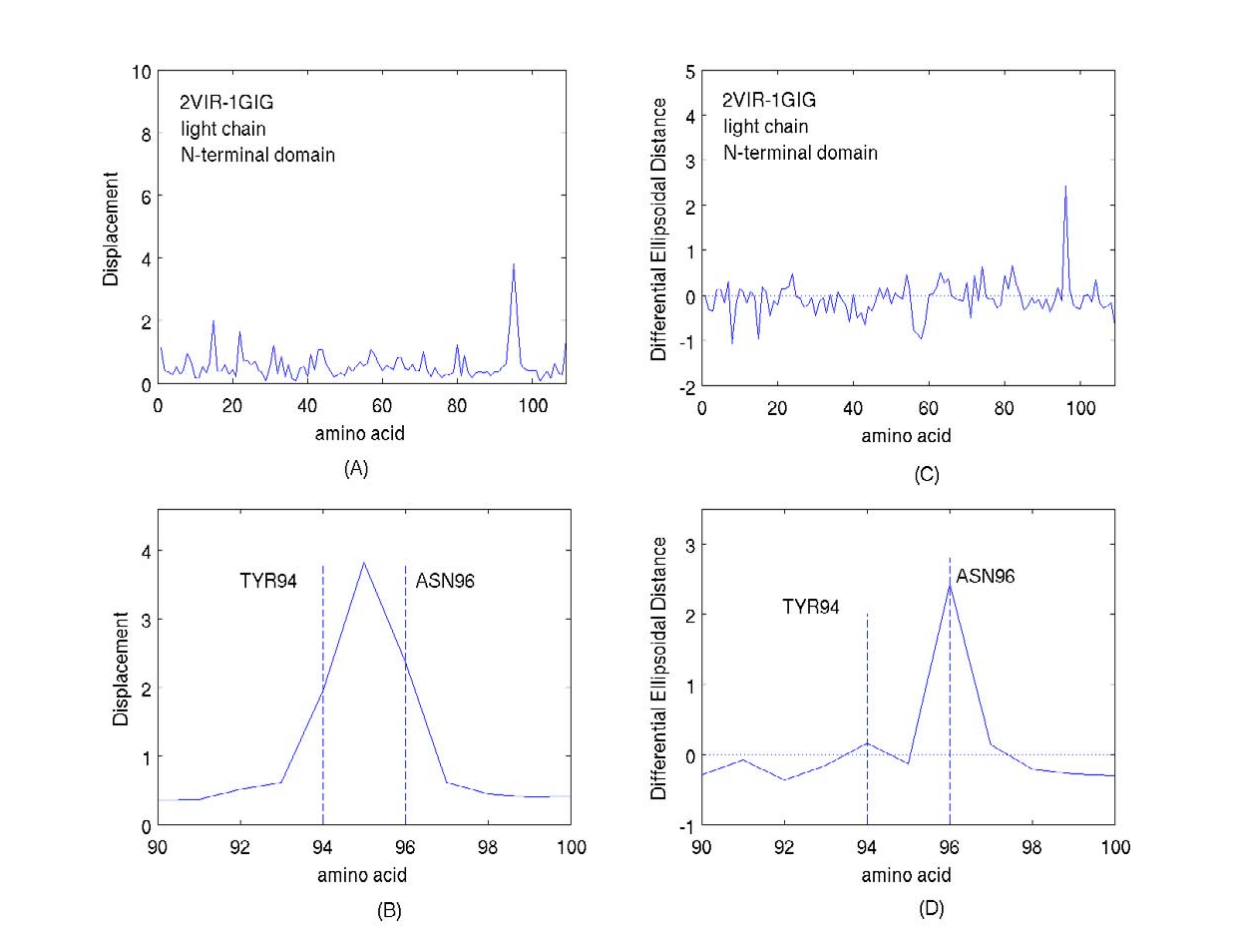
A. The Displacements of the amino acid centroids from their locations in the unliganded domain of the light chain (PDB file, 1GIG) to their locations in the complexed domain of the light chain (PDB file, 2VIR). B. The Displacements described in A, shown on an expanded scale about the amino acid residues of greatest Displacement. C. The differences in the ellipsoidal distances (Differential Ellipsoidal Distances) of the amino acid centroids of the light chain domain of the complex (PDB, 2VIR) from their values in the uncomplexed light chain domain (PDB, 1GIG). D. The differences described in C, shown on an expanded scale about the amino acid residues showing the greatest differences.

Figure 5 shows the displacements and the differential ellipsoidal distances obtained for the amino acids of the light chain. The ordinate scales of figures 5A and 5C have been chosen with the same extent as those of figures 1A and 1C. The smaller displacements and differences of the light chain compared with those of the heavy chain highlight the weaker binding of the antigenic epitope to the light compared with the heavy chain. Examination of the bound structure shows the antigenic epitope to be at a much greater distance from the light chain than its distance from the heavy chain. Aside from the amino acids bracketed by the dashed lines, the displacements and differences in ellipsoidal distances are small; less than 1 Angstrom on average. The bracketed amino acids include TYR94 to ASN96. A number of close distances between the heavy atoms of the amino acid ASN96 of this group and those of SER159 of the "HA-top" are apparently responsible for the interactions that contribute to the enhanced values of the displacements of this group of residues. Furthermore, the lack of correspondence between the magnitudes of the displacements and differential ellipsoidal distances for all of the residues, and in particular for the residues TYR94 and SER95 of this set, is observed and this once again emphasizes the complementary nature of the information provided by these two different spatial metrics.

**Figure 6:**
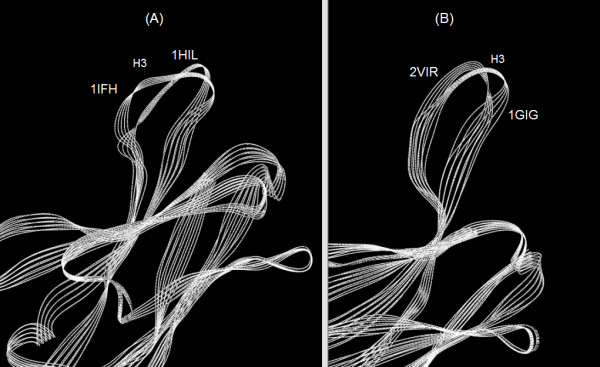
A CE (Combinatorial Extension) superposition of A. the H3 loop of the complexed 1IFH PDB structure upon the H3 loop of the uncomplexed 1HIL PDB structure. B. the H3 loop of the complexed 2VIR PDB structure upon the H3 loop of the uncomplexed 1GIG PDB structure.

A few words should be devoted to the relationship between the intent of the previous X-ray studies and the objectives of the present study. The later X-ray study [6] had focused on the effect of two mutant antigens in inducing structural distortions of the bound complex that could be responsible for the evasion of antibody neutralization. An earlier rigid-body docking study [12] had examined the effect of a number of mutations upon HC19 antibody binding. The present study does not address this issue and has focused solely upon the 2VIR structure; namely, the complex involving the wild type antigenic hemagglutinin "top". The earlier study [8] involved an extensive examination of the structural differences that occur due to antibody binding. Differences in the binding to three different antigenic mimetics of HA1 were examined. It was emphasized that the information obtained was *encouraging for attempts to design synthetic vaccines on the basis of X-ray structures of anti-body-peptide complexes *[8]. It was also stated that *only by comparing the free, unliganded structure with its complexed form is it possible to asses the extent and contribution of conformational changes to the antigen recognition process*; a statement supportive of the strategy of the present work. Furthermore, it was stated that, *Although many Fab Structures have been determined as complexes, only a few have also been described in their uncomplexed state*; a situation which is paralleled to this date since there are, presently, many fewer uncomplexed immunoglobulin structures than complexed structures. Finally, extreme differences in the extent of the conformational adaptations in antibodies as a consequence of antigenic binding had been noted [13], and, *a large conformational change observed in the H3 loop between the free and bound form *[8] was found. This large conformational change of the H3 CDR loop of the 1IFH structure contrasts significantly with the corresponding change of the H3 CDR loop of the 2VIR structure. This difference is illustrated in figure 6 by a CE (Combinatorial Extension) superposition [14] of the H3 antibody loops of the 2VIR and 1IFH PDB complexed structures, upon their respective H3 loops of the1GIG and 1HIL PDB uncomplexed structures. A similar superposition had been previously performed for the CDR loops of the anti HIV Fab 50.1 [15]. The relatively greater distortion of the H3 loop of 1IFH structure compared with that of 2VIR is consistent with the idea that *shape complementarity ...for the smaller, flexible peptides can more easily achieve closer contact with the paratope surface *[16]. Such relatively large structural change of the N-terminal heavy chain domain of the1IFH structure upon binding will be shown to require modification of the present procedure to properly represent the observed structural changes. This provides an example of how the substructures of a set of structures may be selected to obtain sets of relatively invariant principal axes to be used for structural comparison.

**Figure 7:**
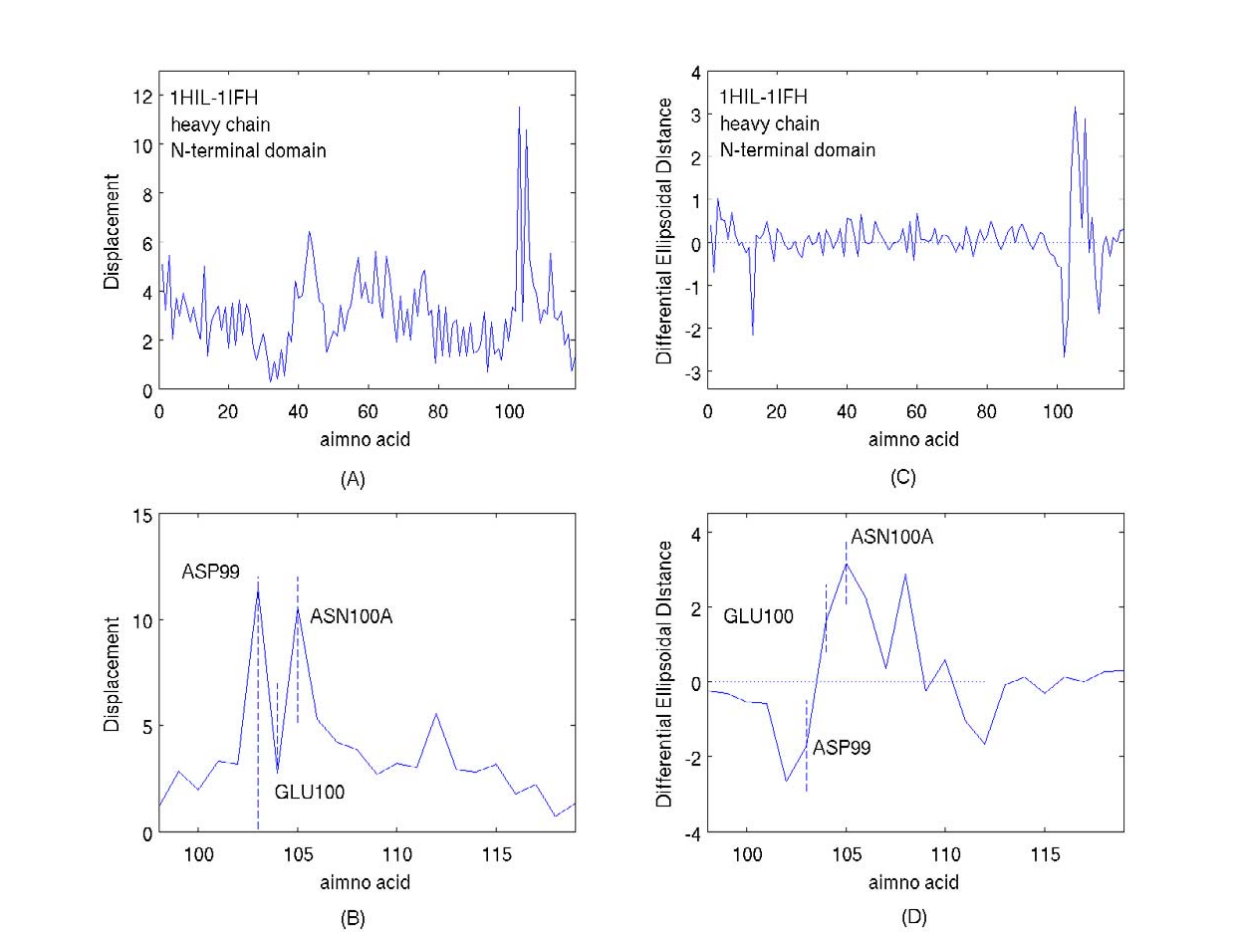
A. The Displacements of the amino acid centroids from their locations in the unliganded domain of the heavy chain (PDB file, 1HIL) to their locations in the complexed domain of the heavy chain (PDB file, 1IFH). B. The Displacements described in A, shown on an expanded scale about the amino acid residues of greatest Displacement. C. The differences in the ellipsoidal distances (Differential Ellipsoidal Distances) of the amino acid centroids of the heavy chain domain of the complex (PDB, 1IFH) from their values in the uncomplexed heavy chain domain (PDB, 1HIL). D. The differences described in C, shown on an expanded scale about the amino acid residues showing the greatest differences.

**Figure 8:**
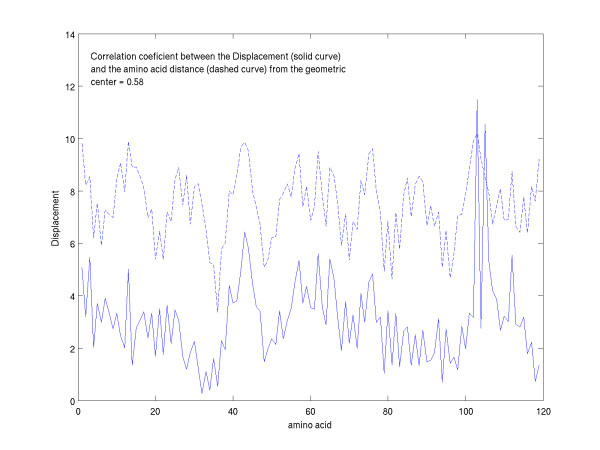
The ellipsoidal distances (dashed) of the antibody heavy chain amino acids (PDB id. 1HIL) overlaid upon their displacements (solid) (PDB id. 1IFH) upon complexing.

Figure 7 shows the displacements and differences in the ellipsoidal distances for the N-terminal domain of the complexed heavy chain of 1IFH with respect to the unliganded heavy chain of 1HIL. It should be noted that the offset in numbering of the abscissa with respect to the residue number is due to the way the amino acid insertions are labeled in the PDB files. The abscissa lists all of the residues in a sequential numbering scheme independent of how they are labeled in the PDB files. A comparison of figure 7A with figure 1A was initially surprising. While one observes significant displacements of those antibody residues interacting directly with the residues of the antigenic mimetic, one also notes that the displacements of the amino acid residues that do not directly interact with the antigen are greater than what had appeared in figure 1A. For example, the mean displacement of the first 95 N-terminal heavy chain amino acid residues of 1IFH-1HIL is 2.94 Angstroms with a standard deviation of 1.32 Angstroms, whereas the mean displacement and standard deviation of the first 95 N-terminal heavy chain residues of 2VIR-1GIG are 0.84 Angstroms and 0.44 Angstroms, respectively. For the 1IFH-1HIL comparison, this appears to belie the original assumption that the majority of the residues that are not interacting directly with the protein mimetic should be minimally displaced from their location in the unliganded structure. Such displacements of up to or greater than 5 Angstroms are observed in figure 7A. This can occur if the two sets of principal axes chosen for comparison are significantly rotated and/or translated with respect to each other when referenced to a global coordinate set of axes. Since the orientation of the axes are obtained by diagonalizing a matrix that is quadratic in the distance of the residues from the center-of-geometry of the domain, a major contribution to such relative rotation would arise from the residues that are most distant from the geometric center of the domain and most significantly displaced. Such correlation between the displacements and distances from the center of the domain is shown visually in Figure 8 where the residue ellipsoidal distances (dashed curve) of 1HIL (a relative measure of distance from the centroid of the domain) are overlaid upon the displacements (solid curve) shown in figure 7A.

Such difference in the orientation of the two sets of principal axes can be significantly reduced by determining the axes for substructures from which significantly displaced distant residues from the center of the domain have been eliminated. While there is a degree of freedom in the choice of such elimination and one may be motivated to optimize the correspondence between the two sets of principal axes used for comparison, the substructures presently chosen will simply involve the elimination of only the two residues ASP99 and ASN100A from the H3 CDR loops, namely, the residues that exhibit the greatest displacements shown in figures 7A and 7B. With the principal axes obtained for both reduced liganded and unliganded substructures one would then rotate the original sets of residue centroids eliminated in the determination of the substructure, into the substructure principal axis orientations after translations to the substructure centers-of-geometry.

**Figure 9:**
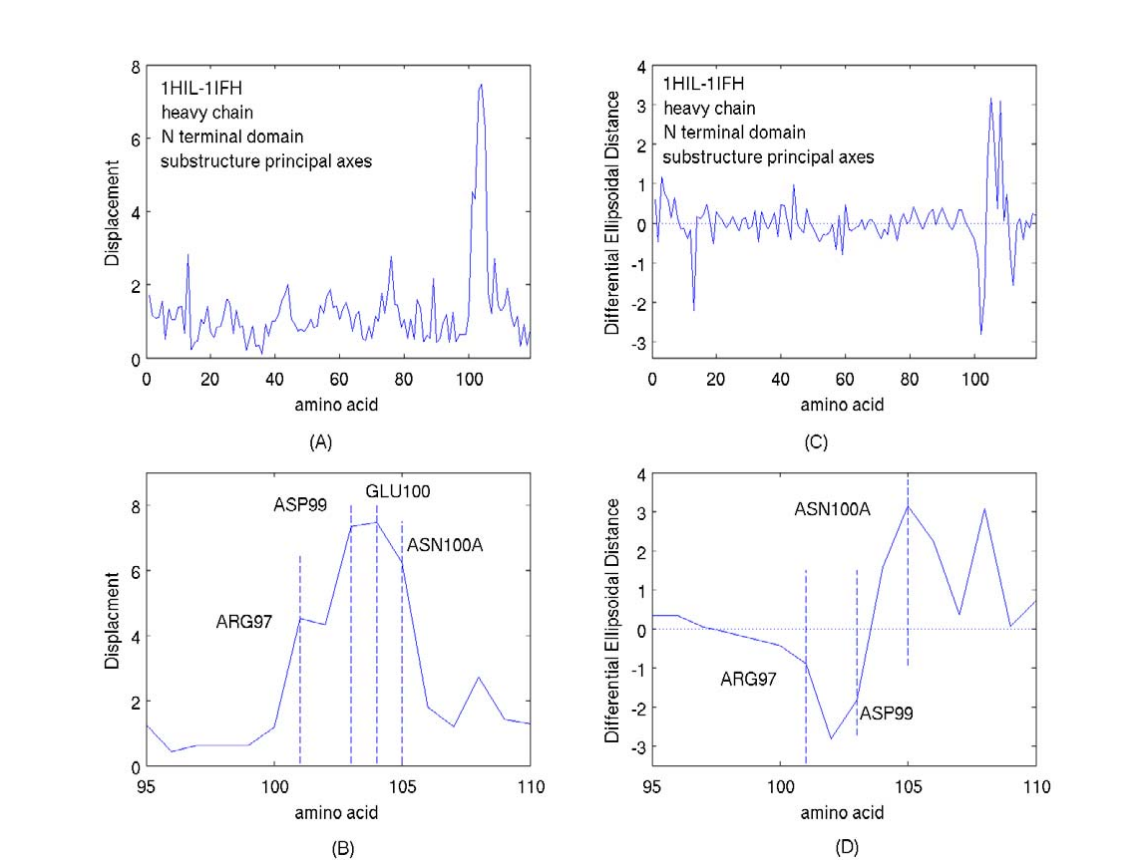
The following quantities obtained after elimination of ASP99 and ASN100A from the substructures determining the principal axes used for comparison. A. The Displacements of the amino acid centroids from their locations in the unliganded domain of the heavy chain (PDB file, 1HIL) to their locations in the complexed domain of the heavy chain (PDB file, 1IFH). B. The Displacements described in A, shown on an expanded scale about the amino acid residues of greatest Displacement. C. The differences in the ellipsoidal distances (Differential Ellipsoidal Distances) of the amino acid centroids of the heavy chain domain of the complex (PDB, 1IFH) from their values in the uncomplexed heavy chain domain (PDB, 1HIL). D. The differences described in C, shown on an expanded scale about the amino acid residues showing the greatest differences.

**Figure 10:**
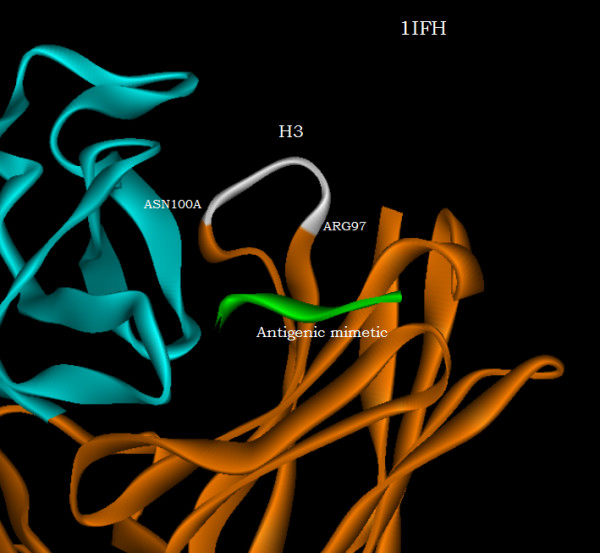
The antibody residues of the 1IFH PDB file highlighted in white that exhibit the greatest displacements upon binding to the antigenic mimetic.

Figure 9 shows the results obtained for the residues of the heavy chain with the residues ASP99 and ASN100A deleted from the substructures determining the principal axes used for comparison. The displacements of residues not directly interacting with the antigen shown in figure 9A are now reduced in magnitude compared with the comparable displacements shown in figure 7A; namely, the first 95 N-terminal heavy chain residue displacements now have a mean of 1.06 Angstroms with a standard deviation of 0.51 Angstroms. Figure 9B shows the greatest residue displacements on an expanded scale, and these residues, ARG97 to ASN100A, near the antigenic mimetic, are highlighted on the terminal loop of the H3 CDR of the antibody shown in figure 10.

Changes in the magnitudes of the ellipsoidal distances contrast with what had been found for the displacements. Comparison of the figures 9C and 7C surprisingly shows comparable ranges of the values of these changes. This is also seen in the expanded scales of figures 9D and 7D which detail the region of interaction and consequently of the region of greatest change. So, one might conclude that the ellipsoidal distances are relatively insensitive to rotations of the principal axes. This is apparently a consequence of the proportionality of the ellipsoidal distances to the radial fractional distances from the center of the domain to the ellipsoidal surface or exterior. Such proportionalities are relatively unchanged as the principal axes are slightly rotated with respect to each other. This would be especially true for a domain approximately spherical in shape.

**Figure 11:**
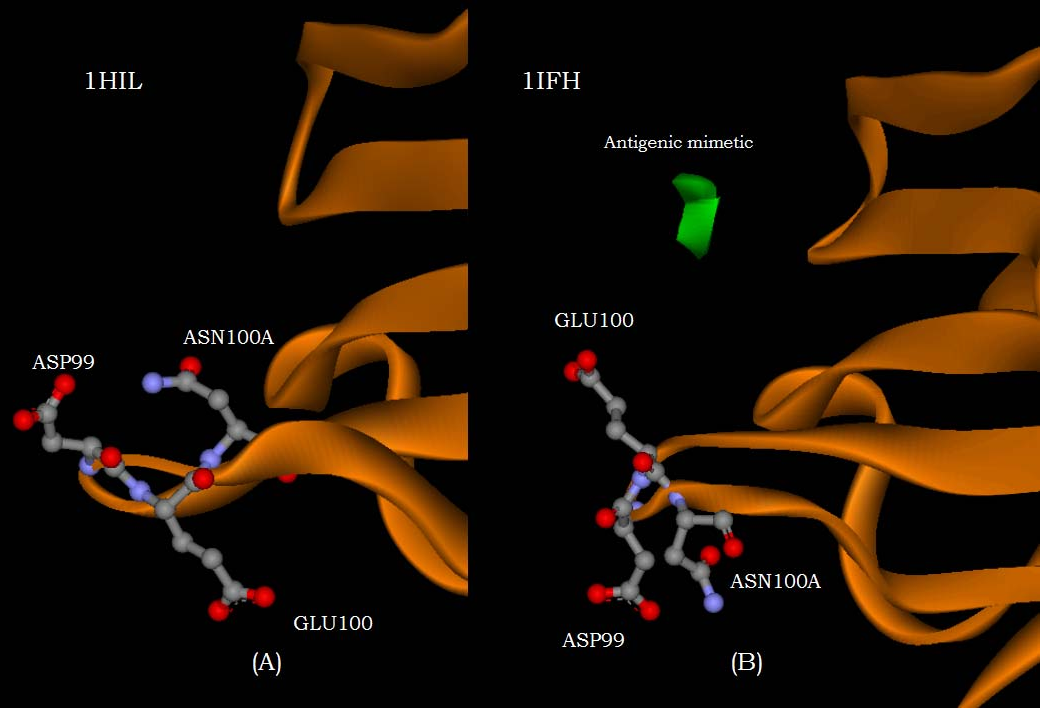
The spatial disposition of amino acids, ASP99, GLU100, and ASN100A of the H3 loop of the heavy chain, A. before (PDB 1HIL) and B. after (PDB 1IFH) complexing with the antigenic mimetic.

Complementary information is again provided by a comparison of the displacements shown in figure 9B with the changes in ellipsoidal distances upon complexing indicated by figure 9D. This comparison shows that while a number of residues of the H3 loop are significantly displaced, some move towards the domain interior of the heavy chain while others move away. Figure 11 is ball and stick representation of three of the residues that are significantly displaced upon complexing. Due to the severe H3 loop distortion upon binding this triplet is rotated from an orientation in which a residue initially pointing either up or down in figure 11A prior to complexing is reversed in direction in figure 11B after complexing. Note, that aside from the H3 loop distortion, the heavy chain orientation has been held relatively fixed in both of the figures. All three of these residues have, therefore, experienced a significant displacement, as shown in figure 11B, from their location prior to complexing. However, while, GLU100 moves away from the center of the N-terminal domain of the heavy chain upon binding, a rotation about its CA-CB bond enhances the motion of the residue centroids, ASP99 and ASN100A, to locations that are respectively, nearer to or more distant from the center of the heavy domain. All of these movements of these three amino acids are summarized simply by the complementary information provided by the two figures, 9B and 9D.

## Conclusion

A new approach, enabling comparison between different, however, structurally related domains, has been applied in determining the structural changes that an antibody protein domain experiences upon its interaction with an antigenic target. The present procedure, while analogous to previous procedures that utilize common symmetry elements for comparison, utilizes, instead, the sets of principal axes of the relatively invariant global structures or substructures of the domains undergoing comparison. An ellipsoidal characterization of these structures yields two spatial metrics that provide complementary information; one, detailing the magnitude of the residue displacements and the other; their direction of their displacement with respect to either the domain exterior or interior. The information provided by the present procedure should augment related information provided by more customary procedures. Hopefully such information will contribute to the *attempts to design synthetic vaccines on the basis of X-ray structures of anti-body-peptide complexes *[8].
